# Misc-GAN: A Multi-scale Generative Model for Graphs

**DOI:** 10.3389/fdata.2019.00003

**Published:** 2019-04-25

**Authors:** Dawei Zhou, Lecheng Zheng, Jiejun Xu, Jingrui He

**Affiliations:** ^1^Arizona State University, Tempe, AZ, United States; ^2^HRL Laboratories, LLC, Malibu, CA, United States

**Keywords:** multi-scale analysis method (MSA), graph generation, generative adversarial network, neural network, cycle consistency

## Abstract

Characterizing and modeling the distribution of a particular family of graphs are essential for the studying real-world networks in a broad spectrum of disciplines, ranging from market-basket analysis to biology, from social science to neuroscience. However, it is unclear how to model these complex graph organizations and learn generative models from an observed graph. The key challenges stem from the non-unique, high-dimensional nature of graphs, as well as graph community structures at different granularity levels. In this paper, we propose a multi-scale graph generative model named *Misc-GAN*, which models the underlying distribution of graph structures at different levels of granularity, and then “transfers” such hierarchical distribution from the graphs in the domain of interest, to a unique graph representation. The empirical results on seven real data sets demonstrate the effectiveness of the proposed framework.

## 1. Introduction

A graph is a fundamental tool for depicting and modeling complex systems in various domains, ranging from market-basket analysis to biology, from social science to neuroscience. Characterizing and modeling the distribution of a particular family of graphs is essential in many real-world applications. For example, in financial fraud detection, generative models are adopted to produce synthetic financial networks, when the empirical studies need to be conducted by the third parties without divulging private information (Fich and Shivdasani, 2007); in drug discovery and development, sampling from the generic model can facilitate the discovery of new medicines which equip new configurations while preserving the property of the existing medicines (Gómez-Bombarelli et al., 2016); in social network analysis, the distributions on graphs can be used to discover new graph structures and generate evolving graphs (You et al., 2018a).

Generative models of graphs have been studied well for decades. Traditional graph generative models (Erdös and Rényi, 1959; Albert and Barabási, 2002; Leskovec et al., 2010) are usually built upon some structural premises, e.g., heavy tails for the nodes' degree distribution, small diameters, and densification in graph evolution. More recent studies on deep generative models (e.g., Kingma and Welling, 2013; Goodfellow et al., 2014), reveal a surge of research interest in modeling graphs. For example, Liu et al. (2017) proposes a deep model for learning characteristic topological features from the given graphs via generative adversarial networks (GAN); (You et al., 2018a) uses a deep autoregressive model to efficiently learn the complex joint probability of all the nodes and edges from an observed set of graphs.

However, real-world networks typically exhibit hierarchical distribution over graph communities, while the existing graph generative models are either restricted to certain structural premises (Erdös and Rényi, 1959; Albert and Barabási, 2002; Leskovec et al., 2010), or unable to capture the hierarchical community structures over the graphs (Grover et al., 2018; Li et al., 2018; Simonovsky and Komodakis, 2018). Developing graph generative models that can capture not only the low connectivity patterns at the level of individual nodes and edges, but also the higher-order connectivity patterns, i.e., the hierarchical community structures in the given graphs, will significantly improve the fidelity of graph generative models and help reveal more intriguing patterns in various domains. For instance, given an author-collaborative network, research groups of well-established and closely collaborated researchers could be identified by the existing graph clustering methods in the lower-level granularity. While, from a coarser level, we may find that these research groups constitute large-scale communities, which correspond to various research topics or subjects. Moreover, different from image data or text data, a graph with *n* nodes can be represented by *n*! equivalent adjacency matrices with node permutation, which increases the difficulty of training the generative model in the first place.

In this paper, we aim to address the following challenges: (***C*.1**) How to capture the community structures at different levels of granularity and how to generate a unique graph representation that preserves such hierarchical graph structures. (***C*.2**) How to alleviate the high complexity of modeling numerous representations of graphs and how to ensure the fidelity of the proposed graph generative model. To address the preceding challenges, we propose a generic generative model of graphs (*Misc-GAN*) to learn the underlying distribution of graph structures at different levels of granularity. In particular, our proposed framework consists of three key steps. First, it coarsens the input graph into the structured representations of different levels (i.e., granularity). Then, inspired by the success of deep generative models in image translation (Kingma and Welling, 2013; Goodfellow et al., 2014), a cycle-consistent adversarial network (CycleGAN) (Zhu et al., 2017) is adopted to learn the graph structure distribution and generate a synthetic coarse graph at each granularity level. Last, the *Misc-GAN* framework defines a reconstruction process, which reconstructs the graphs at each granularity level and aggregates them into a unique representation.

The main contributions of this paper can be summarized as these three aspects:

1. A novel problem setting which aims to model the complex distribution of community structures at different granularity levels in the real networks.
2. A graph generative model which is capable of modeling hierarchical topology features from a single or a set of observed graphs producing high-quality domain specific synthetic graphs.
3. Extensive experiments and case-studies on seven real-world data sets, showing the effectiveness of the proposed framework *Misc-GAN*.

The rest of this paper is organized as follows. We briefly review some related work in section 2, formally define the multi-scale domain adaptive graph generation problem in section 3 and present the formulation and implementation of our proposed *Misc-GAN* framework in section 4. The empirical studies are conducted in section 5. Finally, we conclude this paper in section 6.

## 2. Related Work

In this section, we briefly review the related studies on the generative adversarial network, multi-scale analysis of graph and cycle consistency.

### 2.1. Generative Adversarial Network

In Goodfellow et al. (2014), the authors propose the generative adversarial networks (GANs) to create a generative model and a discriminative model and compare them with each other in the adversarial setting. The authors denote *P*_*z*_(*z*) to be the prior of the input noise variables *z* and *G*(*z*; θ_*g*_) to represent a mapping to data space, where G is a differentiable function represented by a multi-layer perceptron with parameters θ_*g*_. *G*(*z*) maps the noise variables to data space and it aims to generate samples as genuine as possible. The authors also define *D*(*x*; θ_*d*_) to be another multi-layer perceptron or discriminator distinguishing whether the given samples are drawn from the real-world data set or from the fake data set. *D*(*x*) is the probability of *x* coming from the real-world data set rather than the generated data set. In this min-max game, the discriminator *D* aims to maximize the probability of assigning the correct label to both the real samples and the faked samples generated by the generator *G*, while the generator *G* aims to minimize the probability that the discriminator *D* successfully distinguishes the faked samples from the real samples. The objective of this min-max game is written as:

(1) $$\begin{array}{l} \min_{G}\;\max_{D}V(G,D)={𝔼}_{x\sim P_{data}(x)}[\log D(x)]\\ \;+{𝔼}_{z\sim P_{z}(z)}[\log(1-D(G(z)))] \end{array}$$

More recently, a surge of research interest has been observed in data mining and machine learning communities, with respect to using GANs in various real applications. For example, in Du et al. (2018), the authors proposed the adversarially learned inference model to generate shared representation by matching cross-domain joint distribution; the domain-adaptation works in Ganin et al. (2016), Zhang et al. (2017), and Tzeng et al. (2017) tried to minimize the domain-specific latent feature representations in adversarial settings; some GANs-based approaches have been proposed to minimize the distance between feature distributions, such as Ganin et al. (2016), Zhang et al. (2017), and Tzeng et al. (2017). In this paper, the generative adversarial network is the basis from which to transfer graphs from one domain to another, while the local valuable structures of graphs are preserved.

### 2.2. Multi-scale Analysis of Graphs

Multi-scale analysis of graphs has been studied for years in machine learning with wide applications in numerous areas, such as simplification and compression of graphs (Cour et al., 2005; Safro and Temkin, 2011), dynamics of graphs at different resolutions (Lee and Maggioni, 2011; Gao et al., 2016), graph visualization (Stolte et al., 2003), recommendation systems (Gou et al., 2011) and so on. The common assumption of multi-scale analysis is that the given data in a high dimensional space has a much lower dimensional intrinsic geometry. Take the document text as an example, the dependencies among words constrain the distribution of word frequency in a lower dimensional space. Diffusion wavelets (Coifman and Maggioni, 2006) is one common method used in multi-scale analysis which allows us to construct functions on the graph for statistical learning tasks by producing coarser and coarser graphs at different resolution levels. In this paper, we adopt the concept of multi-scale analysis to capture the local structure of graphs at different resolution levels and then reconstruct the graph while preserving these important local structures.

### 2.3. Cycle Consistency

The concept of cycle consistency has been applied to various computer vision problems, including image matching (Huang and Guibas, 2013; Zhou et al., 2015), co-segmentation (Wang et al., 2013, 2014), style transfer (Zhu et al., 2017; Chang et al., 2018), and structure from motion (Zach et al., 2010; Wilson and Snavely, 2013). The idea of cycle consistency constrain is utilized as a regularizer in these algorithms, such as cycle consistency loss used in Zhou et al. (2016) and Godard et al. (2017) to push the mappings to be as consistent with each other as possible in the supervised convolution neural network training. Zhu et al. (2017) proposes the Cycle-Consistent generative adversarial network to learn two mappings or generators *G*:*X* → *Y* and *F*:*Y* → *X* between two domains *X* and *Y*. The authors introduce two adversarial discriminators *D*_*X*_ and *D*_*Y*_, where *D*_*X*_ aims to distinguish the images *x* drawn from the real data set *X* from the fake images generated by *F*(*Y*); similarly, *D*_*Y*_ aims to distinguish the images *y* drawn from data set *Y* from the fake images generated by *G*(*X*). In this paper, we apply this concept to find the graph transfer mappings between domain *X* and domain *Y*, such that the transferred graph from domain *Y* to domain *X* is sufficiently similar to the graph in domain *X*.

## 3. Problem Definition

In this section, we introduce the notation and problem definition of this paper. The main symbols and notations are summarized in Table 1. We use ordinary lowercase letters to denote scalars, boldface lowercase letters to denote vectors, and boldface uppercase letters to denote matrices and tensors. Moreover, the elements (e.g., entries, fibers and slices) in a matrix or a tensor are represented in the same way as the Matlab, e.g., *M*(*i, j*) is the element at the *i*th row and *j*th column of the matrix ***M***, and ***M***(*i*, :) is the *i*th row of ***M***, etc.

**Table 1:** Symbols and notations.

**Symbol**	**Definition and description**
*G*_*s*_, *G*_*t*_	The source domain graph and the target domain graph
${\tilde{G}}_{t}$	The generated graph of the target domain
**A**_*s*_, **A**_*t*_, **Ã**_*t*_	The adjacency matrices of *G*_*s*_, *G*_*t*_ and ${\tilde{G}}_{t}$
*V*_*s*_, *V*_*t*_	The sets of nodes in *G*_*s*_ and *G*_*t*_
*E*_*s*_, *E*_*t*_	The sets of edges in *G*_*s*_ and *G*_*t*_
$G_{s}^{\left(l\right)},G_{t}^{\left(l\right)}$	The induced *l*-th granularity coarse graphs of *G*_*s*_ and *G*_*t*_
*n*_*s*_, *n*_*t*_	Number of nodes in *G*_*s*_ and *G*_*t*_
*m*_*s*_, *m*_*t*_	Number of edges in *G*_*s*_ and *G*_*t*_
*L*	Number of granularity levels
$F^{\left(l\right)},B^{\left(l\right)}$	The generators in the forward and backward GAN at the *l*-th layer
$D_{F}^{\left(l\right)},D_{B}^{\left(l\right)}$	The discriminators in the forward and backward GAN at the *l*-th layer

**Figure 1:**
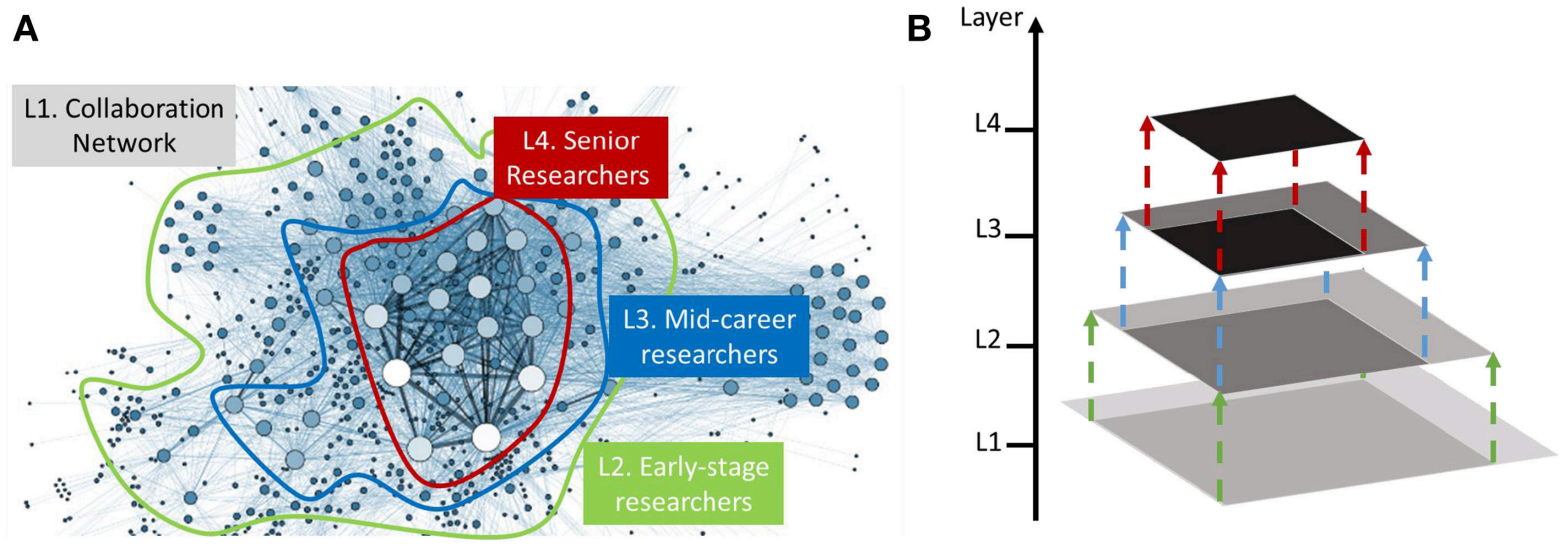
An illustration example. **(A)** Presents a visualization of the collaboration network (Grandjean, 2016). **(B)** Shows the hierarchical structure of the research communities, from early-stage researchers to mid-career researchers and senior researchers.

The goal of this paper is to generate a synthetic target domain graph ${\tilde{G}}_{t}$, by learning mapping functions between the source domain graph *G*_*s*_ and the target domain graph *G*_*t*_. Without loss of generality, in this paper, we assume that a universal structure distribution *p*_*data*_ exists, which defines the structural role of each entity, i.e., node, edge, and subgraph, of the observed graphs. Many existing graph generative models (Bojchevski et al., 2018; You et al., 2018b) are designed to learn the structure distribution of *G* at a single scale, and therefore they might overlook some intriguing patterns in the underlying networks, e.g., the multi-level cluster-within-cluster structures (Ravasz and Barabási, 2003). Figure 1 presents an illustrative example of the hierarchical structures in collaboration networks. In particular, the graph exhibits four-level hierarchies including (L1) all the entities in the collaboration network, (L2) early-stage researchers, (L3) mid-career researchers and (L4) senior researchers. It is unclear how to characterize such hierarchical structures and generate domain-specific synthetic graphs. Moreover, the generative model needs to be scalable when modeling large-scale networks that have exponentially many representations. In this paper, we aim to learn a graph generative model that can automatically translate any source-domain graph into the target-domain graph while preserving the hierarchical structure distribution over the observed target graph. In real cases, the source-domain graphs are sensitive and hard to obtain, while only the target-domain graphs are available to the analysts. For example, in financial fraud detection, the source-domain graphs could be the online transaction network that contains sensitive information (e.g., bank account, personal identification information, transaction amount, etc.); the target-domain graphs that are available to the analysts could be outdated data (transaction data 100 years ago) or the simulated graphs with user-defined graph statistics (e.g., the number of nodes, the number of edges). Thus, by learning such generative model, the third-party analysts can study the data without divulging the sensitive information. With the above notations and objects, we formally define our problem as follows:

Problem 1. **Multi-level Structure-Preserving Graph Generation Input:** (i) a target domain graph *G*_*t*_ = (*V*_*t*_, *E*_*t*_), (ii) a source domain graph *G*_*s*_ = (*V*_*s*_, *E*_*s*_), (iii) the number of granularity levels *L*.

**Output:** (i) a mapping function F that can translate any source-domain graphs to the corresponding target domain graphs while preserving the hierarchical structure distribution over the observed target graph *G*_*t*_, (ii) a generated synthetic target domain graph ${\tilde{G}}_{t}$.

## 4. Proposed Framework

**Figure 2:**
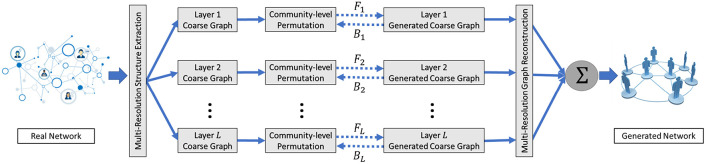
The proposed *Misc-GAN* framework.

In this section, we present our multi-scale graph generative model *Misc-GAN*, which simultaneously characterizes and models the structural distribution of the observed graphs at multiple scales. In particular, we first formulate our framework into a generic optimization problem, and then discuss the details on three modules, i.e., multi-scale graph representation module, graph generation module, and graph reconstruction module, in our proposed framework Figure 2.

### 4.1. A Generic Joint Learning Framework

To address the proposed problem of multi-level structure-preserving graph generation, our joint learning framework should primarily focus on the following aspects. First (*problem setting*), the existing methods are mainly restricted to a single granularity level of graph structures, which might increase the possibility of overlooking the hierarchical community structures in the observed graphs. Thus, the graph generation model should be able to capture the community structures at multiple levels of granularity and generate a unique graph representation. Second (*graph generation performance*), it is unclear how to alleviate the high complexity and ensure the fidelity of the graph generation. This is crucial especially if the observed graphs are noisy and large-scale. With these objectives in mind, we propose a generic graph generation framework as an optimization problem with the following objective function:

$$\begin{array}{l} L=L_{ms}+L_{F}+L_{B}+L_{cyc}\\ \;=\underset{L_{ms}\text{: multi-scale reconstruction loss}}{\underset{︸}{KL(\sum_{l=1}^{L}w^{\left(l\right)}F^{\left(l\right)}(G_{s}^{\left(l\right)})+b,G_{t})}}\\ \;\underset{L_{F}\text{: forward adversarial loss}}{\underset{︸}{+\alpha \sum_{l=1}^{L}{𝔼}_{G_{t}^{\left(l\right)}\sim P_{data}(G_{t}^{\left(l\right)})}[\log D_{F}^{\left(l\right)}(G_{t}^{\left(l\right)})]+{𝔼}_{G_{s}^{\left(l\right)}\sim P_{data}(G_{s}^{\left(l\right)})}[\log(1-D_{F}^{\left(l\right)}(F(G_{s}^{\left(l\right)})))]}}\\ \;\underset{L_{B}\text{: backward adversarial loss}}{\underset{︸}{+\beta \sum_{l=1}^{L}{𝔼}_{G_{s}^{\left(l\right)}\sim P_{data}(G_{s}^{\left(l\right)})}[\log D_{B}^{\left(l\right)}(G_{s}^{\left(l\right)})]+{𝔼}_{G_{t}^{\left(l\right)}\sim P_{data}(G_{t}^{\left(l\right)})}[\log(1-D_{B}^{\left(l\right)}(B^{\left(l\right)}(G_{t}^{\left(l\right)})))]}}\\ \;\underset{L_{cyc}\text{: cycle consistency loss}}{\underset{︸}{+\gamma \sum_{l=1}^{L}{𝔼}_{G_{s}^{\left(l\right)}\sim P_{data}(G_{s}^{\left(l\right)})}[||B^{\left(l\right)}(F^{\left(l\right)}(G_{s}^{\left(l\right)}))-G_{s}^{\left(l\right)}{||}_{1}]+{𝔼}_{G_{t}^{\left(l\right)}\sim P_{data}(G_{t}^{\left(l\right)})}[||F^{\left(l\right)}(B^{\left(l\right)}(G_{t}^{\left(l\right)}))-G_{t}^{\left(l\right)}){||}_{1}]}} \end{array}$$

where the objective consists of four terms. The first term *L*_*ms*_ is the multi-scale reconstruction loss, which is designed to minimize the Kullback-Leibler (*KL*) divergence (Moreno et al., 2004) between the target graph *G*_*t*_ and the generated graph ${\tilde{G}}_{t}$, i.e., ${\tilde{G}}_{t}=\overset{L}{\underset{l=1}{\sum }}w^{\left(l\right)}F\left(G_{s}^{\left(l\right)}\right)+b$. We generalize the conventional *KL* divergence to our problem setting to compare two graphs as follows

(2) $$KL({\tilde{G}}_{t},G_{t})=\sum_{i=1}^{n}\sum_{j=1}^{n}(A_{t}(i,j)+\epsilon )\;\log\frac{A_{t}(i,j)+\epsilon }{{\tilde{A}}_{t}(i,j)+\epsilon }$$

where ***A*_*t*_** and ***Ã*_*t*_** are the adjacency metrics of ${\tilde{G}}_{t}$ and *G*_*t*_, ϵ is a constant with a small value to avoid log(0) or division by 0. The second term $L_{F}$ learns a forward mapping function F from the source graph *G*_*s*_ to *G*_*t*_. The discriminator $D_{F}^{\left(l\right)}$ aims to figure out whether the given graph is a real graph from the target domain or a fake graph generated by the generator F which is transferred from the source domain graph. Similar to the second term, the third term $L_{B}$ defines a backward adversarial loss, which aims to learn the mapping function from the target domain to the source domain. The fourth term $L_{cyc}$ is the cycle consistency loss, which is introduced to further reduce the space of possible mapping function. We argue that learning such bi-directional mapping can largely prevent the learned mapping functions from contradicting each other. At last, we also introduce three positive constants, i.e., α, β, γ, to balance the impact of these four terms in the overall objective function. Follow the min-max scheme of GAN, we aim to solve:

(3) $$F^{*(l)},w^{*(l)},b^{*}=\arg\underset{F^{\left(l\right)},B^{\left(l\right)},w^{\left(l\right)},b}{\min}\underset{D_{F}^{\left(l\right)},D_{B}^{\left(l\right)}}{\max}L,l=1,\ldots ,L$$

### 4.2. Network Architecture

Here, we present our *Misc-GAN* framework (Figure 2). Overall, our framework can be separated into three stages (i.e., modules). In the first stage, our framework takes the input graphs *G*_*t*_ and explores the hierarchical structures by constructing the coarse graphs in *L* levels of granularity (w.r.t. *L* layers in Figure 2). In the second stage, our framework trains an independent graph generative model and produces the multi-scale coarse graph in each layer. In the third stage, our framework autonomously combines the outputs from the previous stage to construct the synthetic graph ${\tilde{G}}_{t}$ that preserves the hierarchical topology features of the given graphs *G*_*t*_.

#### 4.2.1. Multi-Scale Graph Representation Module

In this module, we explore the hierarchical cluster-within-cluster structures in order to better characterize the given graph *G*_*t*_, by using the multi-scale approaches, e.g., hierarchical clustering (Johnson, 1967), algebraic multigrid (AMG) (Ruge and Stüben, 1987). The main idea of AMG-based coarsening is a process of aggregating the strongly coupled nodes with a small algebraic distance to form coarser nodes (Ron et al., 2011). Given a symmetric matrix **G**_*t*_, the coarser graph $G_{t}^{\left(l\right)}$ at the first layer is defined as follows:

(4) $$G_{t}^{\left(1\right)}=P^{(1{)}^{\prime }}G_{t}P^{\left(1\right)}$$

where *P*^(1)^ is a coarser operator for generating $G_{t}^{\left(l\right)}$. This coarser operator follows the weighted aggregation scheme used in Sharon et al. (2000) by assigning the weight to the edge connecting two coarse aggregates, where the weight of $P_{ij}^{\left(1\right)}$ is the fraction of the *ith* node that will belong to the *jth* aggregate. Extending this idea to the *lth* layer, the multi-scale approaches recursively construct a multi-scale hierarchy of increasingly coarser graphs at the *l*-th layer as follows:

(5) $$G_{t}^{\left(l\right)}=P^{(l-1{)}^{\prime }}\ldots P^{(1{)}^{\prime }}G_{t}P^{\left(1\right)}\ldots P^{\left(l-1\right)}$$

where *l* = 1, …, *L*, **P**^(1)^, …, **P**^(*l*−1)^ are the coarsening operators, and *G*_*l*_ is the coarse graph at the *l*-th layer. Based on Equation (5), we construct a set of coarse graphs with multiple scales from the target domain graph *G*_*t*_. These coarse graphs will be fed into the following graph generative module in order to learn the hierarchical structures of *G*_*t*_.

#### 4.2.2. Graph Generation Module

It is challenging to learn the underlying structure distribution *p*_*data*_ of the target domain graph *G*_*t*_, as the graph with *n* nodes can be represented by *n*! equivalent adjacency matrices with node permutations (You et al., 2018a). Some recent works have been proposed to tackle this issue. For example, Simonovsky and Komodakis (2018) proposes an approximate graph matching scheme that requires *O*(*n*^4^) operations in the worst case; (You et al., 2018a) develops a tree-structure node ordering scheme, which is based on breadth-first-search (BFS) to reduce the computational complexity. However, these methods may either suffer from the intractable time complexity, or not well preserve the hierarchical structures of the given networks.

Here, we propose a multi-scale graph generation scheme, which models the complex distribution of graph structures over a pyramid of coarse graphs rather than the original graphs. The intuitions are in the following two aspects: (1) directly training from the coarse graphs facilitates the learning process of the generative model, as the coarse graphs serve as the abstractions of the original graphs; (2) this scheme provides the flexibility for the users to decide the granularity-level of the coarse graphs to be learned, which could be attractive when we need to model the large-scale networks. To be more specific, the graph generation module at each layer (shown in Figure 2) can be separated into three steps: First, we partition the graph into multiple non-overlapping subgraphs using state-of-the-art graph clustering methods (Ester et al., 1996; Schaeffer, 2007). Then, based on the detected communities, we generate a set of block diagonal matrices by shuffling community blocks over the diagonals, which are used to characterize the community-level graph structures. At last, the generated block diagonal matrices are fed into an independent graph generative model to generate the synthetic coarse graphs at each layer.

#### 4.2.3. Graph Reconstruction Module

In this stage, we first adopt the multi-scale approaches to reconstruct the graph from coarse to fine. Given a coarser matrix ${\text{G}}_{t}^{\left(2\right)}$ at the second layer, the fine graph ${\tilde{G}}_{t}^{\left(l\right)}$ at the first layer is defined as follows:

(6) $${\tilde{G}}_{t}^{\left(1\right)}=R^{(1{)}^{\prime }}G_{t}^{\left(2\right)}R^{\left(1\right)}$$

where *R*^(1)^ is a reconstruction operator mapping the coarser graph back to the fine graph ${\tilde{G}}_{t}^{\left(l\right)}$. Extending this idea to multiple layers, the multi-scale approaches recursively construct a multi-scale hierarchy of increasingly refined graphs at the *l*-th layer as follows:

(7) $${\tilde{G}}_{t}^{\left(l\right)}=R^{(1{)}^{\prime }}\ldots R^{(l-1{)}^{\prime }}G_{t}^{\left(l\right)}R^{\left(l-1\right)}\ldots R^{\left(1\right)}$$

where *l* = 1, …, *L*, ${\tilde{G}}_{t}^{\left(l\right)}$ is the reconstructed adjacency matrix from the *l*-th layer, and **R**^(1)^, …, **R**^(*l*−1)^ are the reconstruction operators that maps the coarser graph to the fine graph ${\tilde{G}}_{t}^{\left(l\right)}$. After that, all the reconstructed graphs at each layer are in the same scale as the target graph *G*_*t*_, which could be aggregated into a unique one by a linear function, ${\tilde{G}}_{t}=\overset{L}{\underset{l=1}{\sum }}w^{\left(l\right)}{\tilde{G}}_{t}^{\left(l\right)}+b$, where *w*^(1)^, …, *w*^(*L*)^ are the non-negative weights, and *b* is a bias. Compared with the existing graph generative models (Erdös and Rényi, 1959; Albert and Barabási, 2002; You et al., 2018a), the advantages of using such multi-scale graph reconstruction models are twofold. First, by reconstructing the graph form the multiple coarse graphs, the graph generated by *Misc-GAN* naturally preserves the hierarchical community structures at the different levels of granularity. Moreover, we claim that our proposed *Misc-GAN* framework is more scalable than most existing GAN-based graph generative models, as our model provides the flexibility of being trained from the coarse graph of input networks at a user's preferred scale. In particular, given an online transaction network with millions of nodes, the running and space complexity could be intractable for either GAN or CycleGAN to store and perform computations at the original scale of the input graph. Instead, *Misc-GAN* allows end users to learn from a pyramid of the coarse graphs (e.g., ten thousands of nodes) that preserves the key information of community structures but requires less memory and computational resources.

### 4.3. Training Details

We applied the technique of cycleGAN to the transfer graph from one domain to another domain. Different from the density property of images, the adjacency matrix for a graph is much sparser. In our algorithm, two convolution layers are used to capture the hierarchical structure information of the graph. Because the adjacency matrix of a graph is sparser than the dense matrix of an image, we set the size of stride to four, the size of kernels to 4 × 4 matrices, and the number of kernels to 32 for each convolution layer. Then, *k* iterations of ResNet (He et al., 2016) are applied to accelerate the convergence. Finally, two deconvolution layers were used to reconstruct the adjacency matrix with similar settings used in convolution layers.

Second, following the strategy mentioned in Shrivastava et al. (2017) and Zhu et al. (2017), we updated two discriminators with the history of the generated graph ${Ã}_{t}^{\left(l\right)}$ in the *l*-th layer to reduce the vibration of the model. For all the experiments, we set the training iterations to 250. Adam solver (Kingma and Ba, 2014), with a batch size of one, is used to minimize the loss function and all networks are trained with a learning rate of 0.0002 in the tensorflow deep learning framework.

## 5. Experiment

In this section, we demonstrate the performance of our proposed *Misc-GAN* framework on real networks. Moreover, we present a case study to illustrate the effectiveness of *Misc-GAN* in learning the topological features at different levels of granularity.

### 5.1. Experiment Setup

#### 5.1.1. Data Sets

We evaluated our proposed algorithm on seven real-world networks from the Stanford Network Analysis Project (SNAP) (Leskovec and Krevl, 2015). The statistics of data sets are summarized in Table 2. In particular, Email is a communication network, where an edge exists if one person sends at least one email to another person; Facebook is a social network, where each edge represents a social connection between the users in Facebook; Wiki is a voting network, which is used by Wikipedia to elect administrators among the huge contributors; P2P is a file-sharing network, where each node represents a host and each edge represents a connection between hosts; GNU is another Gnutella peer-to-peer file sharing network, which is similar to P2P network; Bitcoin is a who-trusts-whom network that covers the bitcoin trading information on the Bitcoin OTC platform, where each node represents a user and each edge represents the trustfulness between two users; CA is a collaboration network from arXiv, where each node represents an author and each edge represents the collaborations between authors. For different weights in a graph, i.e., Bitcoin graph, we convert the values of edges to binary values in order to feed them to our model.

**Table 2:** Statistics of the network data sets.

**Network**	**Type**	**Nodes**	**Edges**
Email	Directed	1,005	25,571
Facebook	Undirected	4,039	88,234
Wiki	Directed	8,292	14,547,910
P2P	Directed	10,876	39,994
Gnu	Directed	6,301	20,777
Bitcoin	Directed	5,881	35,592
CA	Undirected	5,242	14,496

#### 5.1.2. Comparison Methods

We compared *Misc-GAN* with two random graph models, i.e., Erdös-Rényi (E-R) model (Erdös and Rényi, 1959) and Barabási-Albert (B-A) model (Albert and Barabási, 2002), and one recent deep graph generative model, i.e., GAE (Kipf and Welling, 2016). All the graph statistics are outlined in Table 2. In our setting, the graphs in Table 2 are target domain graphs, and the source domain graphs are generated under a random normal distribution with the same numbers of nodes and edges as the target domain graphs.

#### 5.1.3. Repeatability

All the data sets are publicly available. We will release the code of our algorithms through the authors' website after the paper is published. The experiments are performed on a Windows machine with four 3.5 GHz Intel Cores and 256 GB RAM.

### 5.2. Quantitative Evaluation

**Figure 3:**
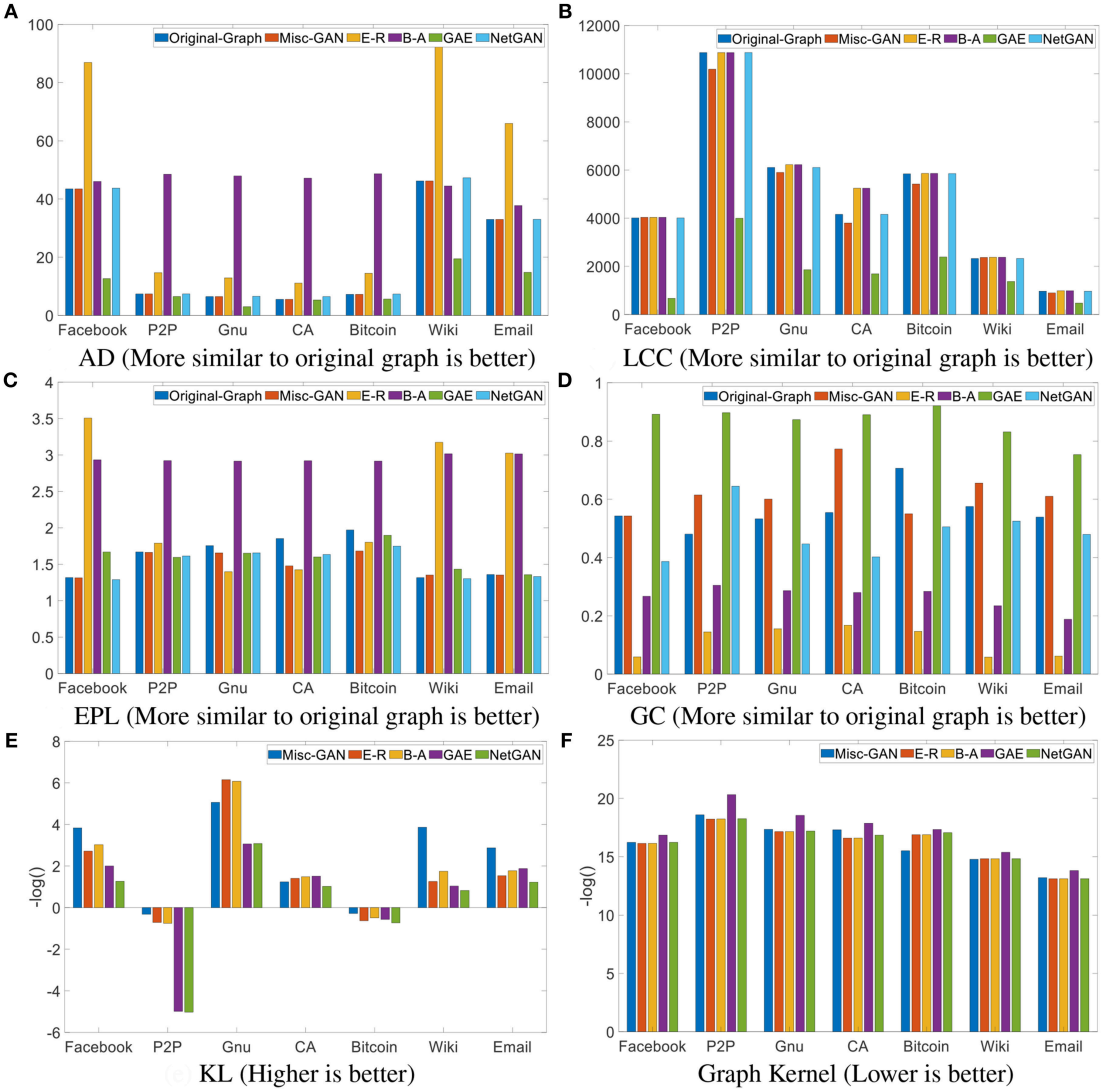
Effectiveness analysis. **(A)** AD (More similar to original graph is better). **(B)** LCC (More similar to original graph is better). **(C)** EPL (More similar to original graph is better). **(D)** GC (More similar to original graph is better). **(E)** KL (Higher is better). **(F)** Graph Kernel (Lower is better).

The comparison results, in terms of effectiveness across a diverse set of real networks, are shown in Figure 3. In particular, we present the results regarding the following metrics: (1) AD: the average degree of all nodes in a graph; (2) LCC: the size of the largest connected component of the graph; (3) EPL: the exponent of the power law distribution of the graph; (4) GC: the Gini coefficient of the degree distribution of the graph; (5) KL: the symmetric Kullback-Leibler (KL) divergence (Moreno et al., 2004) between the local clustering coefficient distributions of the original graphs and the generated graphs; (6) Graph Kernel: the similarity between the original graph and the generated one by using the random-walk based graph kernel (Kang et al., 2012). From these figures, the x-axis of each figure represents a data set, and the y-axis is the value of metrics. From Figures 3A–D, we mainly compare various graph statistics between the original graph and the generated ones using baseline methods. If the value of the metric of the generated graph is close to that of the original graph, it means the generated graph is much more similar to the original graph. We observed that the AD of our proposed algorithm is almost identical to the AD of the original graph for all data sets; for the other three metrics, our proposed algorithm also outperformed the others in most cases. In Figures 3E,F, we present the divergence and similarity score between the original graphs and the generated graphs. Note that, for presentation purposes, all the results in Figures 3E,F are presented using a negative log function, i.e., *f*(*x*) = −log(*x*). In general, we observe that (1) our proposed *Misc-GAN* outperforms the comparison methods across most of the datasets and evaluation metrics in most cases. For example, in the Email data, *Misc-GAN* is 66% smaller on the clustering coefficient distribution evaluation; (2) our proposed *Misc-GAN* framework better preserves the local topological features (e.g., the largest connected component and local clustering coefficient) and the global features (e.g., mean degree, the power law coefficients of the degree distribution of graphs) than other deep generative models (e.g., GAE). It is because our method explores the network structures at multiple resolutions and automatically learns the weight of the importance of topological features at different levels, while the existing deep generative models may fail to model such fine-grained topological features.

**Figure 4:**
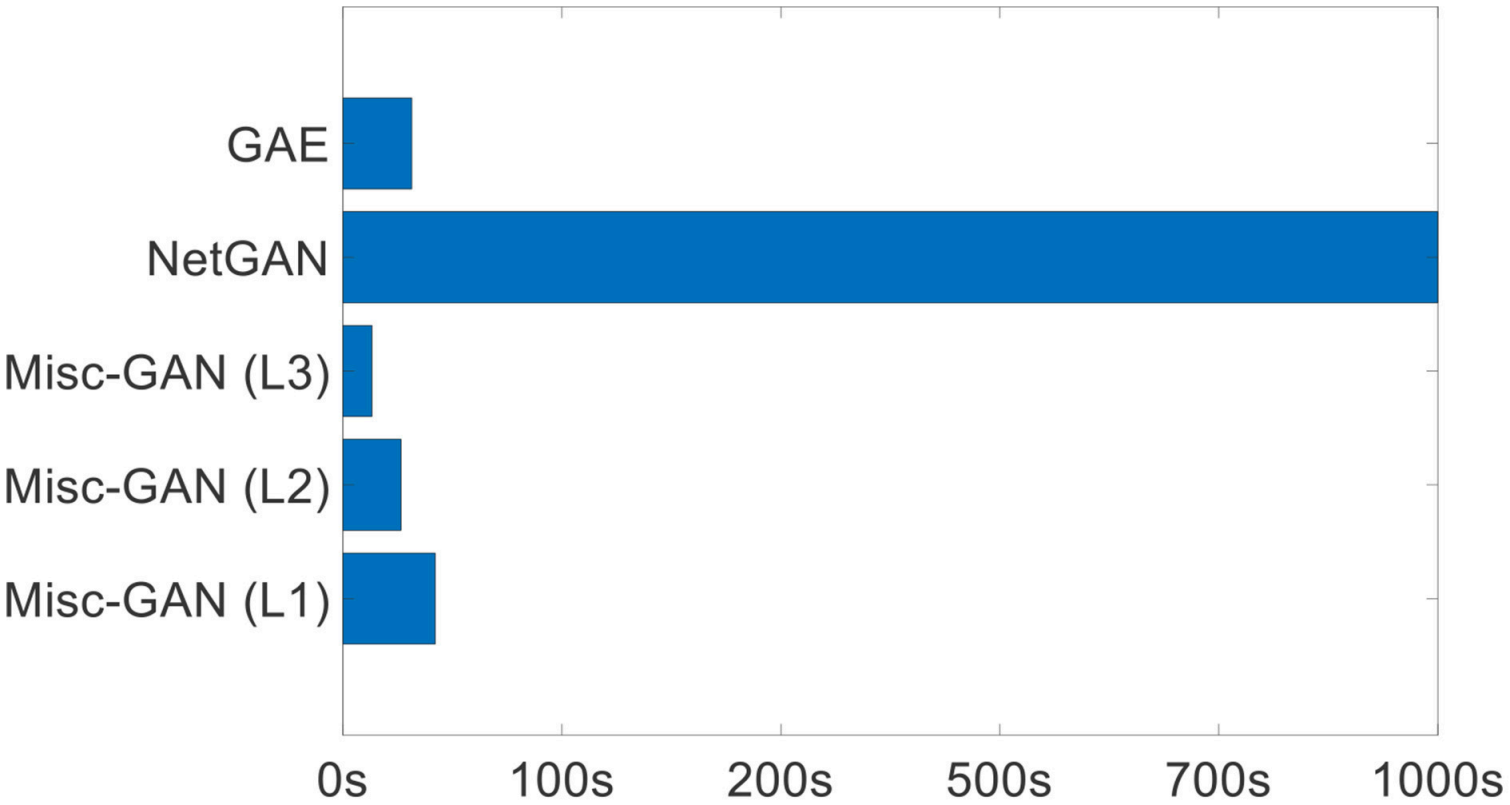
Running time (s) on email dataset.

Moreover, we present the comparison results regarding running time (s) on the Email dataset in Figure 4. In particular, we show the running of *Misc-GAN* learning from the original graph (i.e., *Misc-GAN* (L1)), the Layer 2 coarsen graph with around 700 nodes (i.e., *Misc-GAN* (L2)), the Layer 3 coarsen graph with around 500 nodes (i.e., *Misc-GAN* (L3)), by comparing the two neural network based methods (i.e., GAE and NetGAN). Due to the random graph algorithms (e.g., E-R and B-A) do not have a certain training process, we do not include them in Figure 4. In general, we observed the following: (1) *Misc-GAN* (42s) have comparable running time with GAE (38s), and way faster than NetGAN (1092s); (2) by learning from the Layer 2 coarse graph and Layer 3 coarse graph, the running time of *Misc-GAN* dramatically reduced from 42 s to 26 s and 12 s, respectively.

### 5.3. A Case Study With Respect to the Impact of Multi-scale Analysis

**Figure 5:**
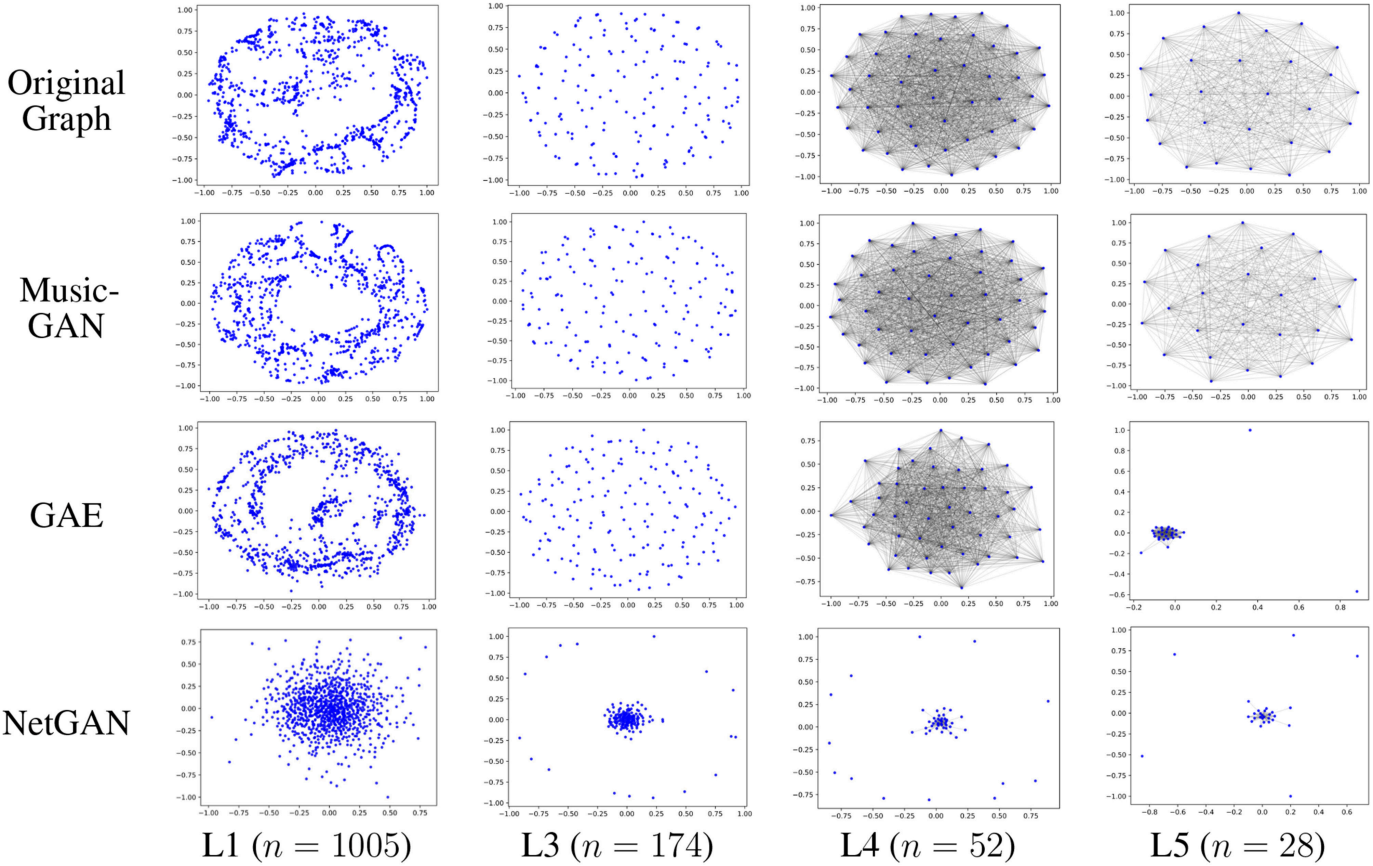
Graph reconstruction at multiple scales.

A simple but intuitive way to evaluate the generated graphs is to visualize the network layout in a two-dimensional space. In Figure 5, we compared the multi-scale network representations of the original graph (i.e., Email) and the generated graphs. In particular, we selected the deep generative model GAE and NetGAN as our baseline methods and constructed coarse graphs at four different scales based on Equation (4). In general, we found that (1) our framework preserved the graph structures at multiple levels of granularity well; (2) NetGAN only preserved the lower-level connectivity patterns (e.g., clusters within a loop pattern) in Layer 1, but failed to capture the higher-level connectivity patterns (e.g., the cluster of super-nodes) in Layer 3, Layer 4 and Layer 5. The reason for the preceding phenomenon is that NetGAN is trained at a single level (i.e., a single granularity of nodes), which results in the coarse reconstruction of high-level network structures. GAE also has a similar problem, due to the failure of capturing higher-level connectivity patterns(i.e., in Layer 5).

## 6. Conclusion

We propose a multi-scale generative model named *Misc-GAN* for graph-structured data, which explores the network structures at multiple resolutions and automatically generates a unique graph representation that preserves such fine-grained topological features. The empirical studies show that *Misc-GAN* achieves significantly better performance than state-of-the-art models do on real networks. However, various challenges remain in this problem, such as how to make the deep generative model scale to massive graphs, and how to generate the domain-specific graph with complex connectivity patterns (e.g., modeling the online transaction networks with money laundering patterns).

## Author Contributions

All authors listed have made a substantial, direct and intellectual contribution to the work, and approved it for publication.

### Conflict of Interest Statement

The authors declare that the research was conducted in the absence of any commercial or financial relationships that could be construed as a potential conflict of interest.
